# Human Fecal Carriage of *Streptococcus agalactiae* Sequence Type 283, Thailand 

**DOI:** 10.3201/eid2908.230098

**Published:** 2023-08

**Authors:** Timothy Barkham, Wen Ying Tang, Yi-Chen Wang, Paiboon Sithithaworn, Kulthida Y. Kopolrat, Chanika Worasith

**Affiliations:** Tan Tock Seng Hospital, Singapore (T. Barkham, W.Y. Tang);; National University of Singapore, Singapore (Y.-C. Wang);; Khon Kaen University, Khon Kaen, Thailand (P. Sithithaworn, K.Y. Kopolrat, C. Worasith)

**Keywords:** *Streptococcus agalactiae*, Group B Streptococcus, carrier, reservoir, source, human, multilocus sequence typing, Thailand, fish, foodborne, bacteria, food safety, streptococci

## Abstract

*Streptococcus agalactiae* (group B *Streptococcus*) sequence type 283 bacteremia, found almost exclusively in Southeast Asia, is associated with consuming raw freshwater fish, but some patients deny consumption. We detected fecal carriage in 5/184 (2.7%) persons in northeast Thailand. Human carriers might contribute to transmission or be the original source of this sequence type.

The epidemiology of the *Streptococcus agalactiae* bacterium (group B *Streptococcus* [GBS]) in Southeast Asia differs from that traditionally seen in the literature. Investigations after the foodborne outbreak of GBS sepsis in 2015 in Singapore concluded that GBS sepsis is primarily a foodborne infection of adults in parts of Southeast Asia (*1*).

The Singapore outbreak was associated with consumption of raw freshwater fish (*2*,*3*). The GBS were serotype III sequence type 283 (ST283) and behaved more aggressively in adults than other GBS; adults without comorbidities made up 22% of the ST283 bacteremia cases but only 2% of non-ST283 GBS cases (*4*). Subsequent studies showed that although ST283 is almost absent from the rest of the world, ST283 disease is widespread around Southeast Asia in humans and tilapia; human data from Laos and Thailand showed ST283 accounted for 76% (Laos) and 73% (Thailand) of invasive GBS collected during 2000–2017 (*1*,*5*). Aquaculture data from Malaysia, Thailand, and Vietnam show that ST283 accounted for 12%–100% of all GBS isolated from streptococcosis in farmed tilapia during 2003–2018 (*1*,*6*). Whole-genome analysis shows ST283 from humans and tilapia are 1 clone (*1*,*7*). Because consumption of raw freshwater fish is common in affected Southeast Asia countries (T. Barkham, unpub. data), raw tilapia could plausibly be the main source of human ST283, although this theory has not been studied.

This fishborne epidemiology is reflected in a report of 2 sisters in their 50s without comorbidities who returned to Laos after visiting friends and relatives in the United States. Both sisters became ill with ST283 bacteremia a day after a meal that included Mekong fish salad, traditionally made with raw freshwater fish (*8*). However, in case–control studies in Singapore in 2015, which reported statistically strong associations between eating raw freshwater fish and ST283 bacteremia, 21/40 participants in a retrospective study (*3*) and 2/9 participants in a prospective study (*2*) denied eating raw freshwater fish. This finding could be because of poor recollection or varying definitions of raw, as in the case of dishes prepared without heat but regarded as being no longer raw (e.g., raw fish fileted or ground and mixed with lemon juice or other sauces). Nevertheless, the possibility of interhuman transmission or that other foods are a source also deserves study. In addition, when authorities investigated a surge of 18 ST283 bacteremia cases in Singapore in July 2020, no affected persons admitted eating raw freshwater fish. Selling raw freshwater fish as a ready-to-eat food was illegal at that time in Singapore, so this denial was not surprising, but it might also suggest an alternative source or vehicle. 

Studies of vaginal and rectal carriage in women in Southeast Asia have not reported sequence types, because their focus has been capsular serotypes for use in developing vaccines. Two small sequencing studies failed to find ST283; 1 looked at stool samples from 82 food handlers and fishmongers in Singapore during the 2015 outbreak (*4*), and the other looked at an opportunistic collection of 38 vaginal GBS samples isolated from women with colpitis in Hanoi, Vietnam, in 2016 (*1*).

Clinical evidence for carriage and human-to-human transmission of this bacterial strain is provided by several cases of ST283 neonatal early onset disease in Hong Kong (*5*) and Laos (*1*); at least 2 from Laos were isolated on the infants’ first day of life, which suggests maternal carriage. We sought to verify carriage. Because ST283 is known to account for 73% of invasive GBS in humans in parts of Thailand (*1*), we assessed ST283 carriage in a population in Thailand that consumes raw freshwater fish, as verified by the widespread occurrence of *Opisthorchis viverrini*, a liver fluke infection acquired by eating raw freshwater fish.

## The Study

We collected samples in January 2019 from Nong Bua and Dong Mun subdistricts of Nong Kung Si District, Kalasin Province, Thailand. This area is within the Northeast region of Thailand, where the prevalence of liver fluke infection is high. Participants were a random sample of persons >15 years of age who had ever eaten uncooked freshwater fish, been infected by or treated for liver flukes, or knew of any family member who had had liver cancer. The study was approved by the Ethics Committee of Khon Kaen University, Thailand (approval no. HE601370). The first feces and urine samples of the morning were chilled and transported to the laboratory at Khon Kaen University. Each fecal sample was processed for parasite (fluke) examination by the formalin ethyl-acetate concentration technique, and aliquots were stored at −20°C and sent to Singapore for GBS detection. DNA was extracted with the QIA DNeasy PowerSoil Kit (QIAGEN, https://www.qiagen.com) with an additional wash step. We used PCR to detect serotype III GBS (*9*) and performed multilocus sequence typing (MLST) (*10*) on DNA extracts that were positive for serotype III. We included an internal control. We centrifuged each urine sample and kept the supernatant for *O. viverrini* antigen analysis (*11*).

We recruited 184 participants; 18 of the 184 stool samples were positive for serotype III GBS. MLST of these 18 samples found five ST283, three ST1, three ST651, one ST17, one ST862, and two with undefined profiles (with alleles 9,1,170,1,1,53,2, and 16,1,2,1,9,2,2); 3 had inadequate PCR products for sequencing. All samples were negative for PCR inhibition. Stool microscopy found fluke eggs in 4 (2.2%) samples. The *O. viverrini* urine-antigen test was positive in the same 4 samples and in 1 additional sample. We detected 1 serotype III GBS sequence type in each of those 5 stool samples: ST283, ST1, ST17, ST651, and 1 sequence without adequate PCR products for MLST.

## Conclusions

This study demonstrates human carriage of GBS ST283 and establishes that humans might play a part in the transmission of ST283 or could even be the original source. Our finding supports the assumed transmission from mother to newborn described in Laos (*1*). Questions remain as to whether carriage is temporary, perhaps reflecting recent dietary exposure, or long lasting. The transmission of *Opisthorchis* flukes depends on human fecal waste being disposed of in fresh water and humans consuming raw fish from the same water, so each species consumes raw products from the other. The prevalence of those flukes in parts of Southeast Asia is a testament to the ongoing presence of these practices, which could explain the high rates of GBS ST283 in humans and tilapia in the same geographic areas. This hypothesized transmission cycle has not been studied for ST283.

The data reported here might not be representative of all the numerous different ethnic and cultural groups in and around Southeast Asia. We hope these data will stimulate and support further epidemiologic studies, including studies on patterns of consumption of raw freshwater fish in different cultural groups in Southeast Asia, as recommended in the ST283 risk profile published by the Food and Agriculture Organization of the United Nations (*12*).

In summary, we detected GBS ST283 in 2.7% of 184 stool samples collected in northeastern Thailand from a population known to consume raw freshwater fish. We remain uncertain of the dynamics of human carriage of GBS ST283 and its contribution to human-to-human transmission, human disease, and the contamination of aquaculture, but our findings indicate that human carriers might play a part in transmitting GBS ST283 or could be its original source. 
